# Citizen science data reveal regional heterogeneity in phenological response to climate in the large milkweed bug, *Oncopeltus fasciatus*

**DOI:** 10.1002/ece3.10213

**Published:** 2023-07-10

**Authors:** Alexis Garretson, Tedra Cuddy, Alexandra G. Duffy, iNaturalist Citizen Scientists, Rebecca E. Forkner

**Affiliations:** ^1^ Graduate School of Biomedical Sciences Tufts University Boston Massachusetts USA; ^2^ The Jackson Laboratory Bar Harbor Maine USA; ^3^ Forensic Science Program George Mason University Fairfax Virginia USA; ^4^ Department of Biology Brigham Young University Provo Utah USA; ^5^ Department of Biology George Mason University Fairfax Virginia USA

**Keywords:** citizen science, large milkweed bug, life‐history, macroecology, mating phenology

## Abstract

Regional populations of geographically widespread species may respond to different environmental factors across the species' range, generating divergent effects of climate change on life‐history phenology. Using thousands of citizen science observations extracted from iNaturalist and associated with corresponding temperature, precipitation, elevation, and daylength information, we examined the drivers of adult mating and of nymphal phenology, development, and group size for populations of the large milkweed bug, *Oncopeltus fasciatus*, in different ecoregions. Research‐grade iNaturalist images were correctly identified 98.3% of the time and yielded more than 3000 observations of nymphal groups and 1000 observations of mating adults spanning 18 years. Mating phenology showed distinct regional patterns, ranging from year‐round mating in California to temporally restricted mating in the Great Lakes Northeastern Coast ecoregion. Relative temperature increases of 1°C for a given daylength expanded the mating season by more than a week in western ecoregions. While increases in relative temperature delayed mating phenology in all ecoregions, greater winter precipitation advanced mating in the California ecoregion. In the eastern ecoregions, nymphal phenology was delayed by increases in summer rainfall but was advanced by relative temperature increases, whereas in western regions, relative temperature increases delayed nymphal phenology. Furthermore, accumulated growing degree days (AGDD) was a poor predictor of developmental progression, as we found a positive but weak correlation between AGDD and age structure only for the Appalachian Southeast North America and the Great Lakes Northern Coast ecoregions. These complex phenological responses of *O. fasciatus* are just one example of how populations may be differentially susceptible to a diversity of climatic effects; using data across a species' whole distribution is critical for exposing regional variations, especially for species with large, continental‐scale ranges. This study demonstrates the potential of photodocumented biodiversity data to aid in the monitoring of life history, host plant–insect interactions, and climate responsiveness.

## Graphical abstract

Using thousands of research‐grade iNaturalist images including more than 3000 observations of nymphal groups and 1000 of mating adults spanning 18 years, we find distinct regional patterns in the direction and magnitude of milkweed bug phenological responses to climate and daylength. Further, we find that iNaturalist observations were correctly identified 98.3% of the time, underscoring their reliability for future studies of phenology, species interactions, and life history. Our results suggest that climate warming may reduce migratory capacity in this species if temperature increases override daylength cues for reproductive diapause in northeastern populations or host plants shift northward to latitudes where daylength constrains insect phenology.

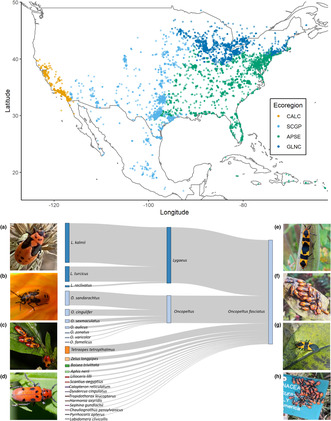

## 1 INTRODUCTION

Phenological alterations in life‐history events are one of the most ubiquitous responses to global climate change. Insect responses include earlier hatching (Kiritani, 2013), better host synchrony (van Asch et al., 2013), and increased voltinism (Altermatt, 2010; Tobin et al., 2008). Resulting changes in insect communities may dramatically alter systems that rely on coordinated timing of reticulate species interaction networks (Beard et al., 2019; Chuine & Régnière, 2017). Unfortunately, broad investigations into climate‐driven changes in insect phenology have not kept pace with the explosive popularity of such studies in plants and vertebrates (Eckert et al., 2010; Lane et al., 2011; Renner & Zohner, 2018; Valenzuela et al., 2019; Visser et al., 2004). Specifically, studies have mainly been limited to adult life‐history stages (Roy & Sparks, 2000), to charismatic taxa like butterflies (Kharouba et al., 2014), or to a few localities or well‐studied regions (Park et al., 2021). These shortfalls prevent the analysis of developmental responsiveness to climate change for most insect taxa and preclude large‐scale predictions for a variety of important factors, such as plant damage, insect outbreaks, or species losses.

Increasing amounts of digital data are readily available to address large‐scale phenological questions for insects. For instance, digitized museum specimens have contributed millions of historical occurrence records (Nelson & Ellis, 2019) used to assess population change or revise range distributions (Halsch et al., 2021; Lemoine, 2015; Wilson et al., 2021), to estimate species phenology (Willis et al., 2017), and to document changes to species interactions (Garretson & Forkner, 2021; Meineke & Davies, 2019). In addition, image‐based citizen science programs are a growing source of data documenting changes in the timing of plant and animal phenophases at a variety of spatial scales (Gerst et al., 2017, 2020; Morisette et al., 2009; Wolkovich & Cleland, 2011). These digitized data also provide developmental information that can be used to investigate species interaction changes and species responses to climate variation on large geographic scales (Belitz et al., 2021; Groom et al., 2021).

Predicting insect phenological change remains complicated despite new data sources. Individual species may respond to minimum, maximum, or variance (extremes) in the daily or seasonal day or night temperatures or precipitation (Bale et al., 2002; Halsch et al., 2021; Kiritani, 2013). Insects' responses to abiotic variables may also interact with static factors, including daylength and topography, to produce complex outcomes (Spence & Tingley, 2020; Yee et al., 2017). Insect phenological responsiveness to climate may also vary within species due to local adaptation (Melero et al., 2022). Phenological differences because of local adaptation may be particularly variable for insects with large historic distributions. Populations across wide geographic ranges may vary in dormancy, migration tendencies, timing or arrival of life stages, and host plant preferences, directly or indirectly generating high heterogeneity in responsiveness to climate. A large geographic distribution may also buffer the impacts of climate change for these species (Bale et al., 2002), or climate variables may have greater predictive capacity across latitude when species span a greater number of ecoregions (Coroian et al., 2014).

The milkweed‐arthropod community provides a model system for investigating large‐scale patterns in the developmental responsiveness of insects to climate change. The milkweed community is highly studied because of the threatened status of the *Asclepias‐*dependent monarch butterfly (*Danaus plexippus*) and the presence of multiple mimicry complexes (Brower, 1958; Duffey & Scudder, 1972). *Danaus plexippus* exhibits one of the most spectacular animal migrations (Brower, 1977; Urquhart, 1976), and its declines have sparked many citizen science programs (Ries & Oberhauser, 2015; Schultz et al., 2017). Since the 1940s, approximately 17% of publications on monarchs have relied on citizen science data (Ries & Oberhauser, 2015). Less attention has been paid to other milkweed‐associated arthropods, despite the importance of diverse and healthy milkweed communities to monarch survival (Stevenson et al., 2021). The large milkweed bug, *Oncopeltus fasciatus* Dallas (Hemiptera: Lygaeidae), is a gregarious *Asclepias* seed feeder found from Southern Canada to Central America. Hemipterans are understudied relative to their species richness (Andrew et al., 2013), but early work on *Oncopeltus* focused on the effects of temperature on life‐history variation (Baldwin & Dingle, 1986; Dingle et al., 1980; Miller & Dingle, 1982). *Oncopeltus fasciatus* populations occupy highly variable climates and microhabitats of plant availability and suitability across this range. Their hemimetabolous life history, in which nymphs often develop into adults on the same plant, allows investigations of subtle impacts of climate change on developmental timing on a continental scale for a single species.

Like monarch butterflies, *Oncopeltus* is a migratory insect. Reproductive diapause induced by colder temperatures and shorter days permits high‐latitude adults to migrate southward. Early laboratory experiments demonstrated the sensitivity of reproductive diapause to temperature and daylength experienced by fifth‐instar nymphs (Dingle, 1974). Specifically, higher proportions of adults enter diapause when fifth instars experience 12 h or less of daylight. Yet nymphs exposed to temperatures ≥27°C may not undergo diapause as adults regardless of daylength, leading to differences in age at first reproduction from as few as 10 days to as much as 102 days (Dingle, 1974). Furthermore, nondiapausing females in these experiments had lower clutch sizes (Dingle, 1974). Temperatures that speed degree‐day‐driven nymphal development and simultaneously expose fifth instars to warmer conditions may override diapause induction, reduce clutch sizes, and interfere with migration. The variation in clutch size in *O. fasciatus* between diapausing and nondiapausing females as well as with temperature (Baldwin & Dingle, 1986) suggests that interactions between daylength and climate change may not only affect diapause and, thus, migration but may also affect nymphal survival, as nymphs experience higher survival in larger feeding aggregations. Furthermore, timing egg hatch and nymphal development to the availability of pods and seeds in this species is critical as nymphs fed vegetative plant parts have stunted growth and adults fail to reproduce (Ralph, 1976). Such life history traits may combine with a wide geographic range to produce complex responses to climate across populations in ecoregions that vary substantially in abiotic factors.

To investigate heterogeneity in climatic responsiveness of adult and nymphal phenology for this wide‐ranging insect, we developed and annotated a large dataset of citizen‐contributed images of *O. fasciatus* on iNaturalist. We (1) estimated the relationship between *O. fasciatus* mating phenology and climatic variables throughout its species range, (2) described the developmental progression of *O. fasciatus* nymphs across ecoregions and elevation, and (3) investigated how nymphal group size varied over ecoregions, with climate variables, and depending on which plant part the nymphs occupied. We further evaluated the quality of the identifications on iNaturalist research‐grade observations of *O. fasciatus*. While studies using citizen science or other novel digitized data have addressed questions of species distribution and range shifts, we illustrate the utility of these information sources to additional aspects of life history and development.

## 2 MATERIALS AND METHODS

### 2.1 Annotation of *Oncopeltus fasciatus* life history

We queried the iNaturalist dataset (Nugent, 2018) for research‐grade images of *O. fasciatus* (https://doi.org/10.15468/dl.hqjp28). Research‐grade observations are those for which at least two‐thirds of community‐provided identifications agree on the species‐level identification. We excluded images with obscured or private coordinates, as well as observations with positional accuracy >100 km. The remaining occurrences (*n* = 12,240) included a geo‐referenced location of occurrence, an observation date, and an image of the insect(s). Next, we excluded images depicting pinned specimens or a specimen in a container or on a hand, images too blurry to count individuals, images that appeared digitally manipulated (e.g., a screenshot from another device), and images in which individuals could not be confirmed to be *O. fasciatus*. We excluded images for which *O. fasciatus* was not the correct identification and updated those observations on iNaturalist accordingly (*n* = 187, see Section 3). In total, this yielded 11,224 records with full spatial and climate data spanning from June 2003 to May 2020. Finally, we used the Similar Species endpoint in the iNaturalist API (https://api.inaturalist.org/v1/docs/) to extract counts of incorrect species‐level identifications on observations later confirmed to be *O. fasciatus*. We used these counts to create a Sankey diagram illustrating the process of correct identification from initial submission to research‐grade confirmation as *O. fasciatus*.

For each of the resulting records, we annotated the first observation image for the number of adults and subadults, the presence of mating adults, and the part of the plant occupied by the insects. For the part of the plant, we assigned images to one of the following based on the location of insects in the image: flowers, open pods, closed pods, stem or branch, leaves, or not on plants (most frequently on the ground or non‐natural structures). We did not identify all host plants because photographs often lacked diagnostic characteristics. For each observation, we extracted the elevation from the Amazon Web Services terrain tiles (Larrick et al., 2020) using the elevatr package in R (version 0.3.1, Hollister et al., 2020). We also extracted the minimum and maximum temperatures and precipitation on the observation date and during the preceding winter from the Daymet gridded climate data using the daymetr package in R (version 1.4 Hufkens et al., 2018; Thornton et al., 2020). We further used the Daymet data to estimate accumulated growing degree days (AGDD) starting from January 1 of the observation year with a lower threshold of 14°C and a maximum threshold of 38°C (Feir, 1974; Lin et al., 1954). Lin et al. (1954) observed a 38°C upper threshold for egg hatch and development in *O. fasciatus*, but no published studies identified nymphal developmental maximum thresholds. The maximum temperature for all nymphal records observed in our dataset was 39°C, so we used 38°C as a conservative upper threshold of nymphal development as well.

### 2.2 Assignment to climate regions

**FIGURE 1:**
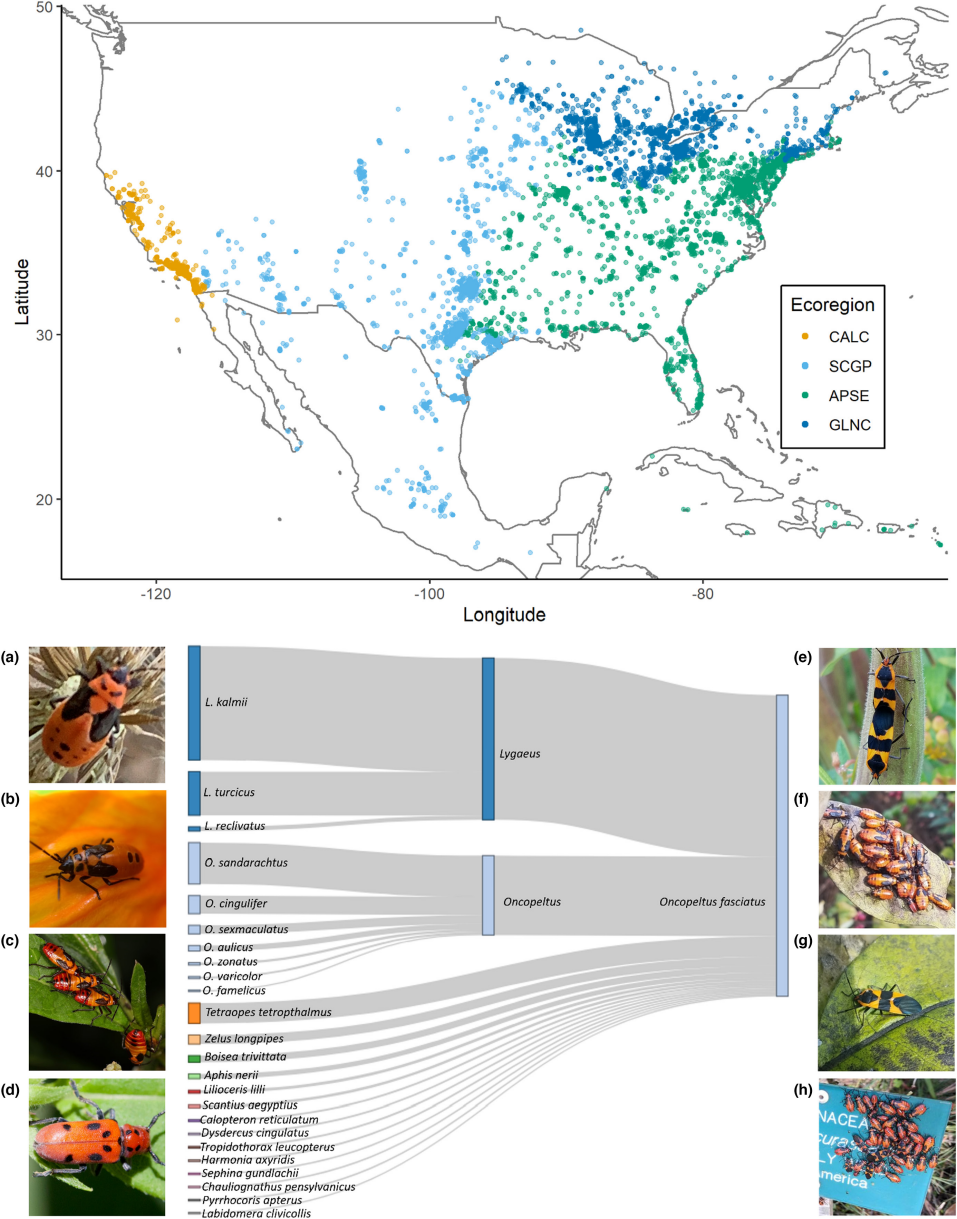
Map locations for all 11,224 iNaturalist observations of *Oncopeltus fasciatus* (top) colored by ecoregions (see Section 2). iNaturalist photos (bottom) on the left are examples of species to which users often first assign *O. fasciatus* observations: (a) *Lygaeus kalmii* nymph by cazotea, (b) *Lygaeus turcicus* nymph by arachnoto, (c) *Oncopeltus sandarachtus* nymphs by Judy Gallagher, (d) *Tetraopes tetrophthalmus* adult by Glenn Berry. The center Sankey diagram shows the proportion of identifications initially incorrectly classified (*n* = 1356), then updated to *O. fasciatus*. iNaturalist photos on the right represent observations of *O. fasciatus*: (e) mating adults on closed pod by iNaturalist user lotteryd, (f) nymphal cluster on leaf, (g) single adult on a leaf, (h) cluster of nymphs and adults on a manmade object by Andy Kleinhesselink.

We categorized observations into ecoregions by location (Figure 1). Ecoregions are areas of similar climate, community characteristics, and *Asclepias* host plant availability. We defined ecoregions based on Level I and Level II ecoregions used by the United States Environmental Protection Agency (USEPA) and based on the Monarch Watch program waystations, accounting for the number and density of observations to ensure a relatively even sample size across regions. Our ecoregions categories were: (A) California Coast (CALC), which includes the Level I ecoregions of Mediterranean California, Marine West Coast Forest, and Northwestern Forested mountains; (B) South Central and Great Plains (SCGP), which includes the Level I ecoregions North American Desert, Southern Semiarid Highlands, Temperate Sierras, Tropical dry forests, and the Great Plains; (C) Great Lakes and Northern Coast (GLNC), which included the Level II ecoregions of the Atlantic Highlands, Central USA Plains, Mixed Wood Plains, Mixed Wood Shield; and (D) the Appalachians and Southeastern North America (APSE), which included the Ozark/Ouachita‐Appalachian Forests, Plain and Hills of the Yucatan Peninsula, Southeastern USA Plains, Everglades, Mississippi Alluvial and Southeastern USA Coastal Plains and the Eastern Temperate forest (split between Appalachian and Northern Forests in Great Lakes).

### 2.3 Analysis of nymphal group size and plant part occupancy

We investigated whether the nymphal group size varied by the part of the plant that the group occupied (see Annotation methods described above). To test whether the trends observed in the models of plant parts were explained by underlying climate variability, we then used analysis of covariance (ANCOVA) to determine whether the average number of subadults in a nymphal group varied significantly across the fixed factors plant part, ecoregion, and their interaction, using elevation, latitude, annual mean temperature, and annual total precipitation as covariates. In fixed factors that were significant in the ANCOVA, we used a post hoc Tukey HSD test to compare the means between groups.

### 2.4 Analysis of mating and nymphal group phenology

To assess how the yearly patterns of mating or the presence of nymphs varied across the *O. fasciatus* range, we fit a Generalized Additive Model (GAM) with day of year as a smoothed predictor to the probability that an iNaturalist image depicted (1) mating insects or (2) nymphal groups (3+ subadults) across each ordinal day of year (DOY). Unlike our model of phenology described below, in which we know a photodocumented occurrence represents a specific date on which that phenophase occurred at that location, our “absences” of this phenology are not “true absences,” and are likely to be biased by the collector behavior (Boakes et al., 2010; Courter et al., 2013). Due to these limitations, we did not generate more complex models of the probability of mating and instead comment on the general trends suggested by the patterns of mating observation. To determine the sensitivity of mating and nymphal phenology to climate variables, we then limited analyses to only observations that depicted mating or nymphal groups, excluding images of individual *O. fasciatus*. Because DOY both dictates daylength and is highly positively correlated with temperature (i.e., in the northern hemisphere, longer days in the summer have higher temperatures and shorter days in the winter have lower temperatures), and because we wanted to account for the influence of temperature on mating season and nymphal development separately from solar insolation, we first regressed maximum daily temperature on daylength using a General Additive Model and daylength as a smoothed predictor for each observation in the full dataset and computed the residuals (Figures S1 and S2). Positive residual values indicate that temperatures were warmer than average for that DOY/daylength and negative values indicate that temperatures were cooler than average for that DOY. Average daylength ranged from 9.1 to 15.6 for observations in our dataset.

With primarily presence‐only data from opportunistically collected data, fitting models to all available phenological presence dates to produce an estimate of mean date is more robust to changes in sampling size over time and space than methods to model first or last dates of phenophase occurrence (Jones & Daehler, 2018). Therefore, to model mean mating date, we fit GAMs for the full dataset and for each ecoregion to the mating occurrence dates as a function of the residual temperature, winter precipitation (mm), and winter maximum temperature (°C), the elevation of the observation, and a smoothed two‐dimensional function of the latitude and longitude (Fang & Feng, 2013; Yee & Mitchell, 1991). We selected winter climate as the relevant season for predicting mating behavior because *O. fasciatus* exhibits facultative reproductive diapause, which is particularly sensitive to declining temperatures and photoperiod (Dingle, 1974; Tauber et al., 1986). To model mean nymphal group dates, we fit GAMs for the full model and for each ecoregion to the occurrence dates as a function of residual temperature, summer precipitation, and summer maximum temperature, elevation of the observation, and a smoothed two‐dimensional function of the latitude and longitude. Contrary to the models of mating adults, we instead selected summer climate as a predictor of nymphal phenology because previous work in this species has demonstrated a close link between ambient temperatures and nymphal aggregations (Barrett & Chiang, 1967; Bongers, 1969). Modeling the latitude and longitude of an observation simultaneously allows models to better account for spatial autocorrelation than linear models and reveal complex patterns of spatial variation in response variables within each ecoregion (Segurado et al., 2006). We fit and graphed models using the mgcv R (version 0.1.6, Wood, 2004, 2011, 2017) and mgcViz (version 1.8–36, Fasiolo et al., 2020) packages.

### 2.5 Analysis of developmental progression

For each nymphal group (3+ subadults), we calculated the proportion of adults in the total number of individuals depicted as a proxy of the developmental progression of the nymphal group. We selected this metric as a proxy for developmental progression because we could not fully assess the developmental stage of subadults and assign them to nymphal instar(L1‐5) developmental stages based on photographs. Further, this metric may be influenced by adult behavior patterns and we are not able to capture initial cluster conditions using these opportunistically collected data. Because of these limitations, we opted for a relatively simple analysis to determine whether we could detect any relationship between the accumulated growing degree days or day of year and elevation for these observations. We calculated the Pearson's R correlation coefficient between AGDD or DOY on the observation date and proportion of adults for each ecoregion. For regions that had significant correlation coefficients, we split observation by elevation (<50 m, 50–200 m, 200+ m) and calculated the correlations between AGDD or DOY and proportions of adults in each elevation category. However, in the supplemental materials, we also provide results for a more complex general linear model with a quasibinomial family and the day of year (or AGDD), elevation, and the interaction term (Table S1). We found that the model fit was quite low, as we expected from the aforementioned sources of additional variation in these data, so include only correlation outputs in the main text.

## 3 RESULTS

### 3.1 Identification

Images depicted an average of 6.63 ± 12.05 (mean ± SD) *O. fasciatus* individuals. Observation photos depicting a single adult were the most common observation type, accounting for 50.58% of our data. For the 11,224 observations, iNaturalist contributors made 22,279 total attempts at species‐level identifications, spanning 24 total taxa. Contributors' initial identifications were incorrect for 1356 (6.1%) of these attempts, of which 53.8% (730) were misidentified as *Lygaeus*, 26.3% (356) misidentified as other *Oncopeltus* species, and 6.8% (92) misidentified *Tetraopes* (Figure 1). Despite incorrect first identifications, within the research‐grade dataset only 1.7% (187) of the observations did not depict *O. fasciatus* and required correction. As with incorrect contributor initial identifications, the majority of the incorrect research‐grade images were corrected to *Lygaeus* species.

### 3.2 Nymphal group size and plant part occupancy

**FIGURE 2:**
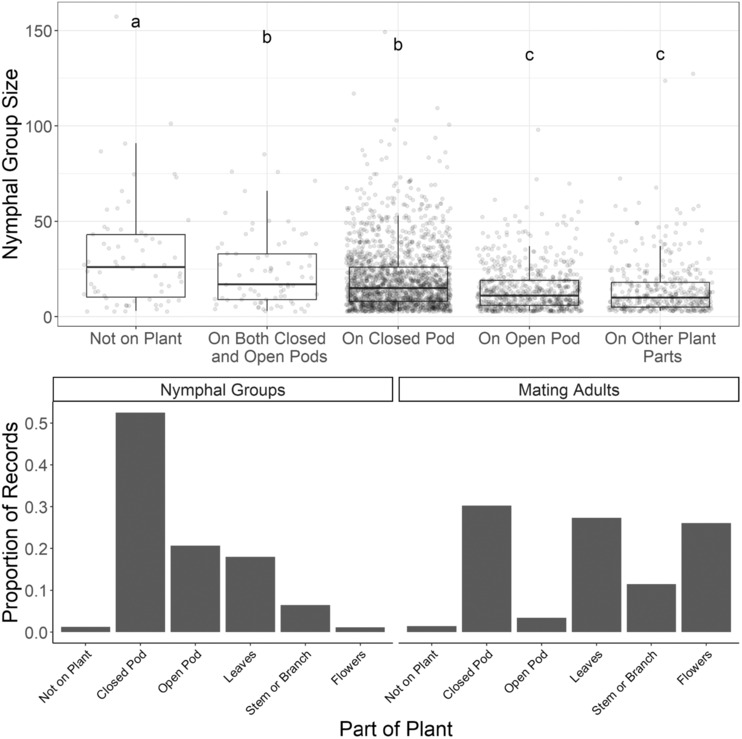
Average nymphal group size (top) for groups of ≥3 individuals for each *Asclepias* spp. plant part. Each point represents an observation and is jittered about the x‐axis to show the density of observations in each group. Letters above box plots denote significant differences across plant parts at *α* = .05 by Tukey's HSD. The proportion of observations (bottom) of nymphal groups or mating adults observed on the different plant parts.

Nymphal groups occurred in 3003 images (26.8%, Figure 2). Occasionally, two or one subadults were photographed alone (4.26%), but these did not meet our criteria for nymphal groups and were excluded from the analysis. The average nymphal group size (the number of subadults within a group) was 18.29 ± 16.21 (mean ± SD). Nymphal group size differed by plant part (Figure 2, *F* = 26.19, *p* < .0001) and across ecoregions (*F* = 4.04, *p* = .0071), although the covariates of maximum temperature, precipitation, elevation, and latitude, as well as the interaction of plant part and ecoregion, were not significant. The largest nymphal groups were those observed off of plants (30.92 ± 3.53), with groups observed on closed pods or a combination of closed and open pods (19.44 ± 0.42 and 22.55 ± 2.16, respectively) greater than those observed on other plant parts (14. 5 ± 0.51, Tukey post hoc comparison, *F* = 3.86, *p =* .05). Nymphal group sizes were higher in the GLNC region (19.41 ± 0.51) compared with the 3 other ecoregions (approximately 2.5 subadults more than the average across other ecoregions; Tukey post hoc comparison, *F* = 3.64, *p =* .05).

### 3.3 Mating phenology

Mating adults occurred in 1006 (9%) of observations (Figure 2). In 6.94% of observations, multiple adults were observed, but they were not mating and were not included in this analysis. Mating adults were observed approximately equally on closed pods (33.2%), leaves (30.1%), or flowers (26.6%) but less frequently on open pods (3.8%). Mating pairs were observed off of plants only 16 (1.6%) times and were observed only 6 (0.6%) times on plants that could be visually confirmed to be non‐Apocynaceae. Notably, one observation documented adults mating on *Erechtites hieraciifolius* (L.) Raf. (Asteraceae), which has not been reported in prior field studies of *O. fasciatus*. All observations of mating on non‐Apocynaceae plants occurred in the southern portions of the range.

**FIGURE 3:**
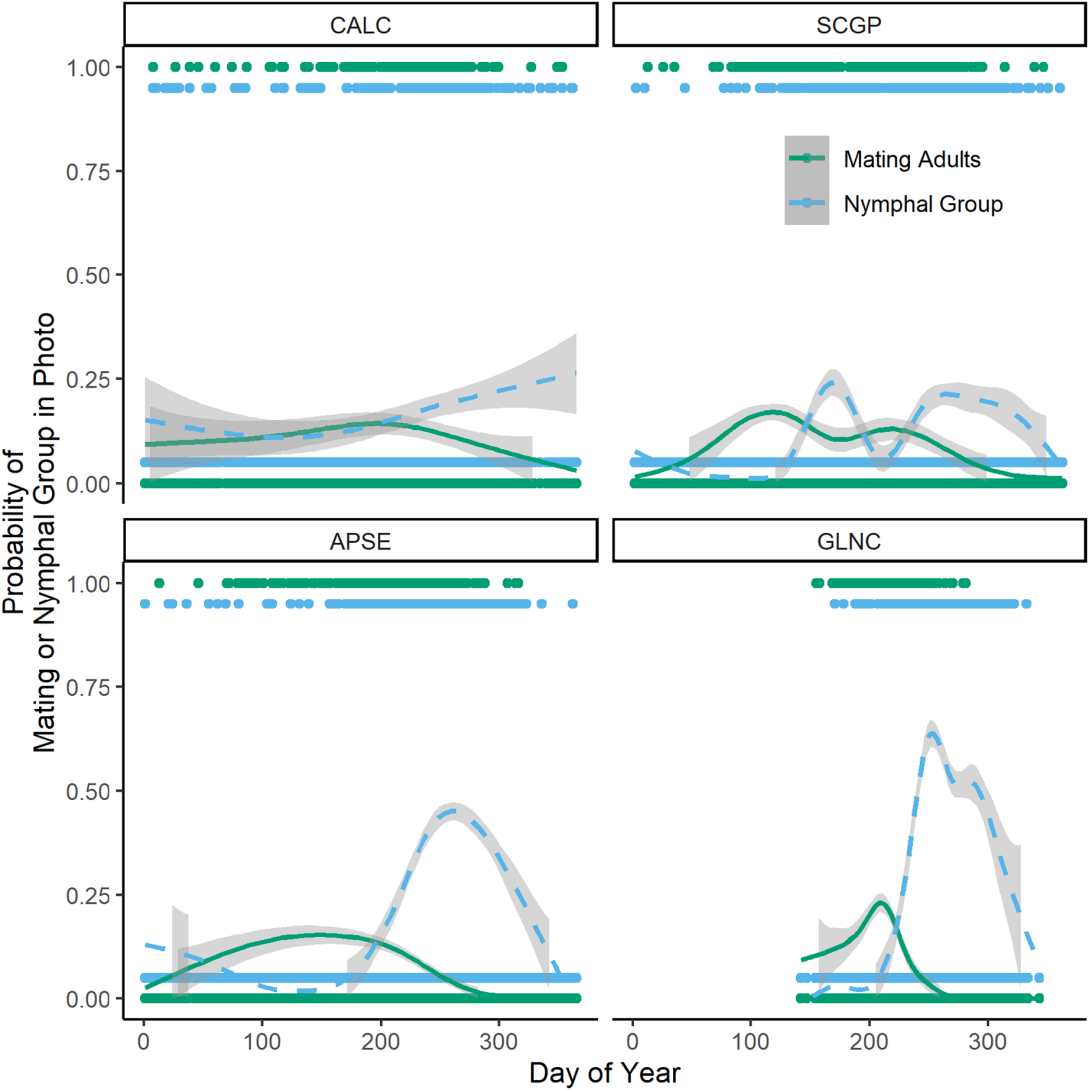
The probability that an observation of *Oncopeltus fasciatus* depicted a mating adult (solid green) or a nymphal group (dashed blue) across the ordinal day of year for the four ecoregions (as in Figure 1). Each point is an observation assigned a 1 (mating adults or nymphal group present) or 0 (mating adults or nymphal group not depicted). Curves are GAM best‐fit curves showing the probability of occurrence across the year for each life stage with gray 95% confidence intervals.

Annual mating phenology of *O. fasciatus* varied across the four ecoregions. The average DOY (mean ± SD) on which mating adults were observed was 201 ± 50 (July 20), 206 ± 64 (July 25), and 217 ± 21 (August 5) for the APSE, CALC, and GLNC ecoregions, respectively (Table 1, Figure 3). Based on best‐fit GAM curves, the CALC ecoregion (Figure 3) exhibited a consistent presence of mating adults with no sharp peak in mating behavior or complete absence (probability value = 0) of mating at any time of year. The SCGP ecoregion, containing observations in the most southern latitudes, showed mating peaks at the end of April (DOY 120.8) and in July (DOY 207.6, Figure 3), giving an average DOY of the mating of more than 40 days earlier than other ecoregions (159 ± 61, June 8). The APSE ecoregion showed a gradual increase in mating adults, with peak mating in July and subsiding by October (Figure 3). Populations in the GLNC ecoregion showed a single, sharp peak in mating behavior in early August, and a steep decline in mating probability, reaching zero probability by mid‐September (Figure 3).

**TABLE 1:** Summary of parameter estimates for generalized additive models (GAM) for mating adult (top) and nymphal (bottom) phenology, including number of observations (*N*), percent deviance explained for model (%Dev Exp), and coefficients (*β* ± SE) for residual temperature (see Section 2), elevation, total winter precipitation, and maximum winter temperature.

Model	Mean DOY ± SD	*N*	% dev exp	Residual temperature	Elevation (m)	Winter Precip (mm)	Winter max temp (°C)	Lat, long (edf)
**Mating adults**								
CALC	206 ± 64	130	22.0%	5.29 ± 1.03***	−0.04 ± 0.02	−0.09 ± 0.024*	−7.56 ± 3.79*	2*
APSE	201 ± 50	254	35.6%	2.52 ± 0.73***	0.01 ± 0.02	−0.04 ± 0.03	−2.98 ± 2.18	2*
SCGP	159 ± 61	279	62.5%	9.85 ± 0.56***	0.08 ± 0.01***	0.04 ± 0.035	1.79 ± 1.23	6.48***
GLNC	217 ± 21	193	27.20%	−1.48 ± 0.55**	−0.08 ± 0.04	0.01 ± 0.04	−2.44 ± 1.29	18.04
FULL	190 ± 57	856	43.30%	5.24 ± 0.38***	0.009 ± 0.010	−0.34 ± 0.02*	−1.96 ± 1.08	19.08***
**Mating Adults**								
CALC	225 ± 72	165	14.70%	5.00 ± 1.23***	0.05 ± 0.02*	−0.01 ± 1.03	−3.10 ± 2.64	2
APSE	222 ± 64	1069	19.40%	−0.71 ± 0.29*	0.01 ± 0.01	0.02 ± 0.01*	0.83 ± 1.43	22.38***
SCGP	253 ± 35	271	26.40%	1.48 ± 0.89	0.02 ± 0.04	0.06 ± 0.03	4.31 ± 4.33	15.81
GLNC	260 ± 21	1083	8.40%	−0.47 ± 0.18*	0.03 ± 0.01*	0.02 ± 0.01*	1.10 ± 0.97	17.81***
FULL	251 ± 40	2588	20.90%	0.31 ± 0.20	0.02 ± 0.005***	0.02 ± 0.01**	0.44 ± 0.64	22.64***

*Note*: Latitude (lat) and longitude (long) were included in the model as smoothed two‐dimensional terms to account for spatial variation (see Figure S1), and estimated degrees of freedom (edf) are provided. * .05 < p > .01, ** 01 < p > .001, *** p < .001.

Generalized Additive Models showed that increases in the amount of precipitation and increases in winter maximum temperatures for the winter prior to collection were significantly associated with advances in the timing of mating for populations in the CALC ecoregions (Table 1). Residual temperature on the collection date was a significant predictor of mating phenology for all ecoregions in our study, but the direction of this effect varied (Table 1). In the majority of ecoregions and in the full model, higher temperatures than average for the given daylength were associated with mating adults occurring at later dates in the season. SCGP, CALC, and APSE populations delayed mating by 9.9, 5.3, and 2.5 days/°C increase in residual temperature, respectively. By contrast, higher residual temperatures on a given day resulted in a 1.5 days/°C advancement of mating times in GLNC. Increases in elevation were associated with later mating only for the SCGP ecoregion.

**FIGURE 4:**
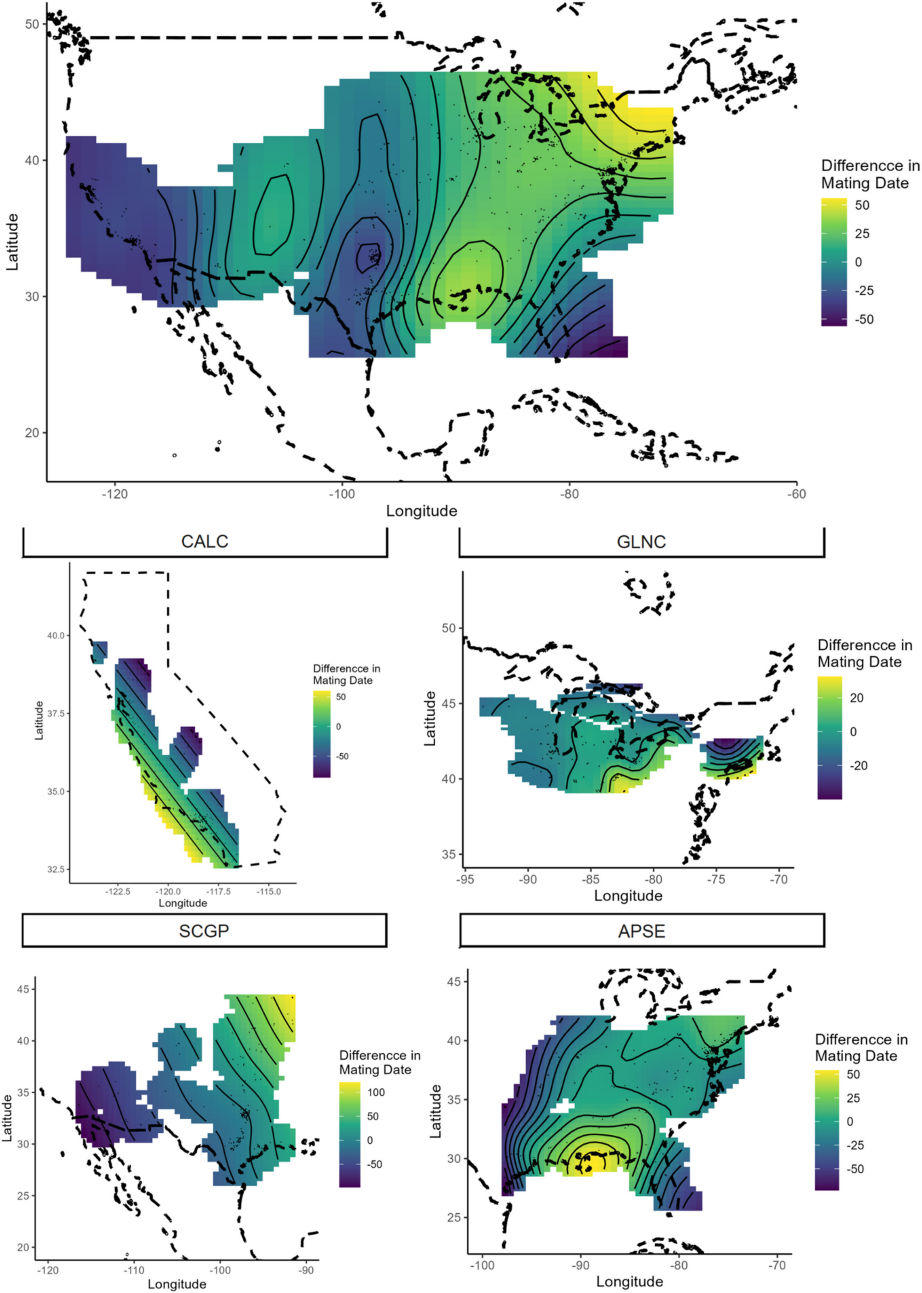
Spatial representation of mean mating occurrence overall (top) and by ecoregion (bottom). Two‐dimensional smoothed curves of latitude and longitude fit simultaneously in GAM. Lighter (yellow) hues indicate delays (later) in the presence of mating or nymphs relative to the mean for that ecoregion, Darker (blue) hues indicate advances (earlier) in mating and nymphs relative to the mean.

Latitude and longitude were significant predictors of the timing of mating for the full model, which indicated that populations in the GLNC mated nearly 100 to 150 days later than populations in the extreme southern portions of the range (southwestern California, south central Texas, and southern Florida, Figure 4). However, when ecoregions were modeled separately, only the SCGP and APSE ecoregions showed significant spatial variation in mating phenology (Figure 4). In the SCGP regions, populations in southwestern California mated more than 150 days earlier than those in the central midwestern states. In the APSE region, populations in southern Florida mated a month or more prior to those in the Appalachian and Blue Ridge Mountain areas.

### 3.4 Nymphal phenology

The average DOY (mean ± SD) that nymphs were observed was 222 ± 64 (August 10), 225 ± 72 (August 13), 253 ± 35 (September 10), and 260 ± 21 (September 17) for the APSE, CALC, SCGP, and GLNC ecoregions, respectively (Table 1, Figure 3). Based on GAM best‐fit curves, the presence of nymphal groups varied across ecoregions in patterns that were similar to but delayed by approximately 1 month (19 to 43 days) from peak adult mating activity (Figure 3). Generalized Additive Models showed that nymphal phenology was associated with the summer precipitation for the eastern ecoregions (APSE and GLNC, Table 1). However, nymphal phenology was not related to maximum summer temperatures in any ecoregion. The residual temperature was associated with nymphal phenology in all ecoregions except SCGP, but in different directions for eastern and western regions: Increasing residual temperature on the day of observation advanced the average date nymphs were observed by 0.71 ± 0.29 and 0.47 ± 0.18 days per residual degree in the APSE and GLNC ecoregions, respectively. This means in the APSE and GLNC ecoregions, warmer temperatures than the average for a given daylength were associated with an earlier appearance of nymphs in those regions. On the other hand, increases in residual temperature (higher temperatures than expected for the daylength) delayed the appearance of nymphs by 5.00 ± 1.23 days per residual degree in the CALC ecoregion.

**FIGURE 5:**
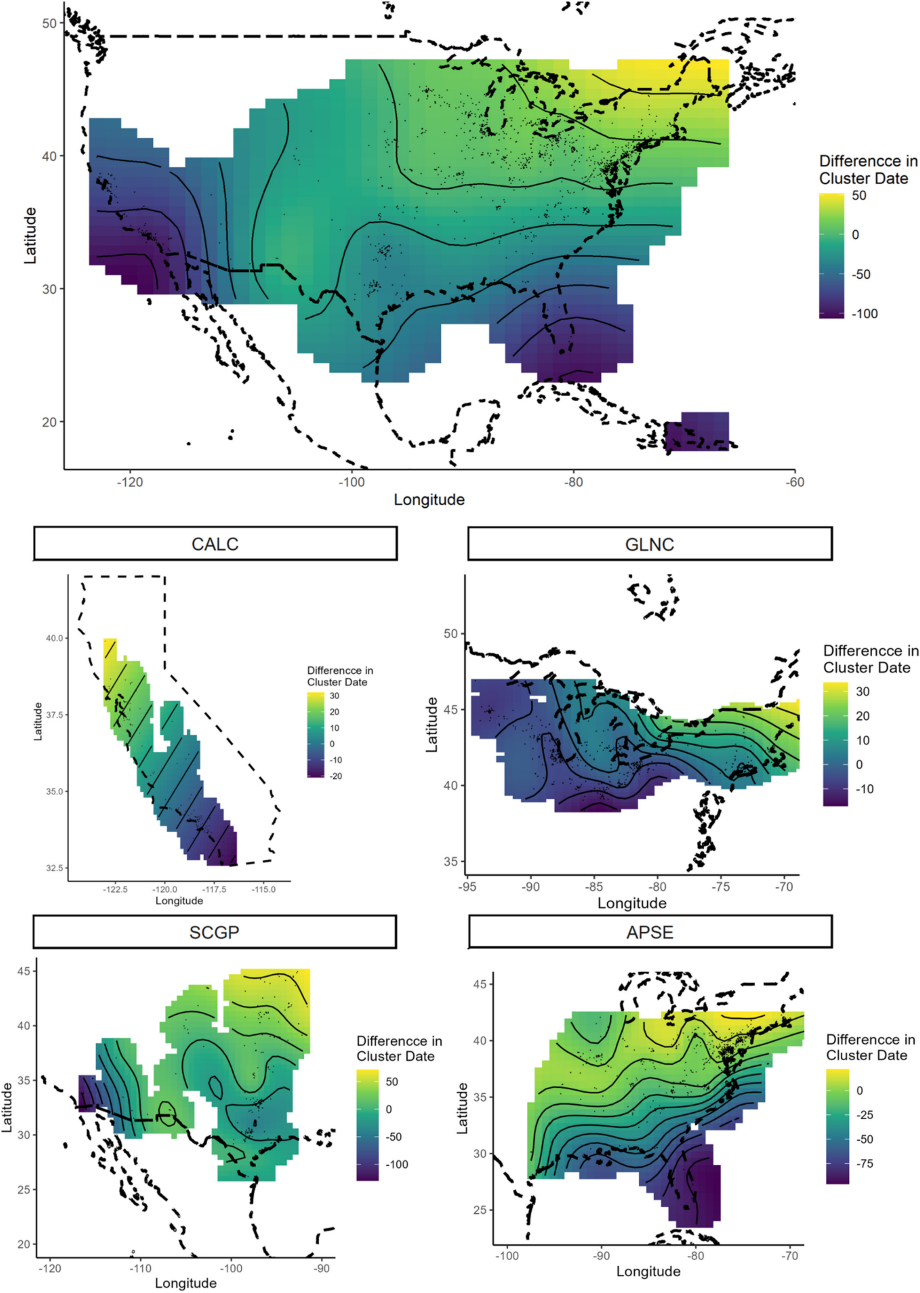
Spatial representation of mean nymphal group occurrence overall (top) and by ecoregion (bottom). Two‐dimensional smoothed curves of latitude and longitude fit simultaneously in GAM. Lighter (yellow) hues indicate delays (later) in the presence of mating or nymphs relative to the mean for that ecoregion, darker (blue) hues indicate advances (earlier) in mating and nymphs relative to the mean.

Patterns of spatial variation in nymphal phenology were similar to those of adults (Figure 5). Latitude and longitude were not significant in models of nymphal phenology in the CALC or SCGP ecoregions (Table 1, Figure 5). Increases in elevation were associated with the later appearance of nymphs for both the GLNC and CALC ecoregions.

### 3.5 Developmental progression

**FIGURE 6:**
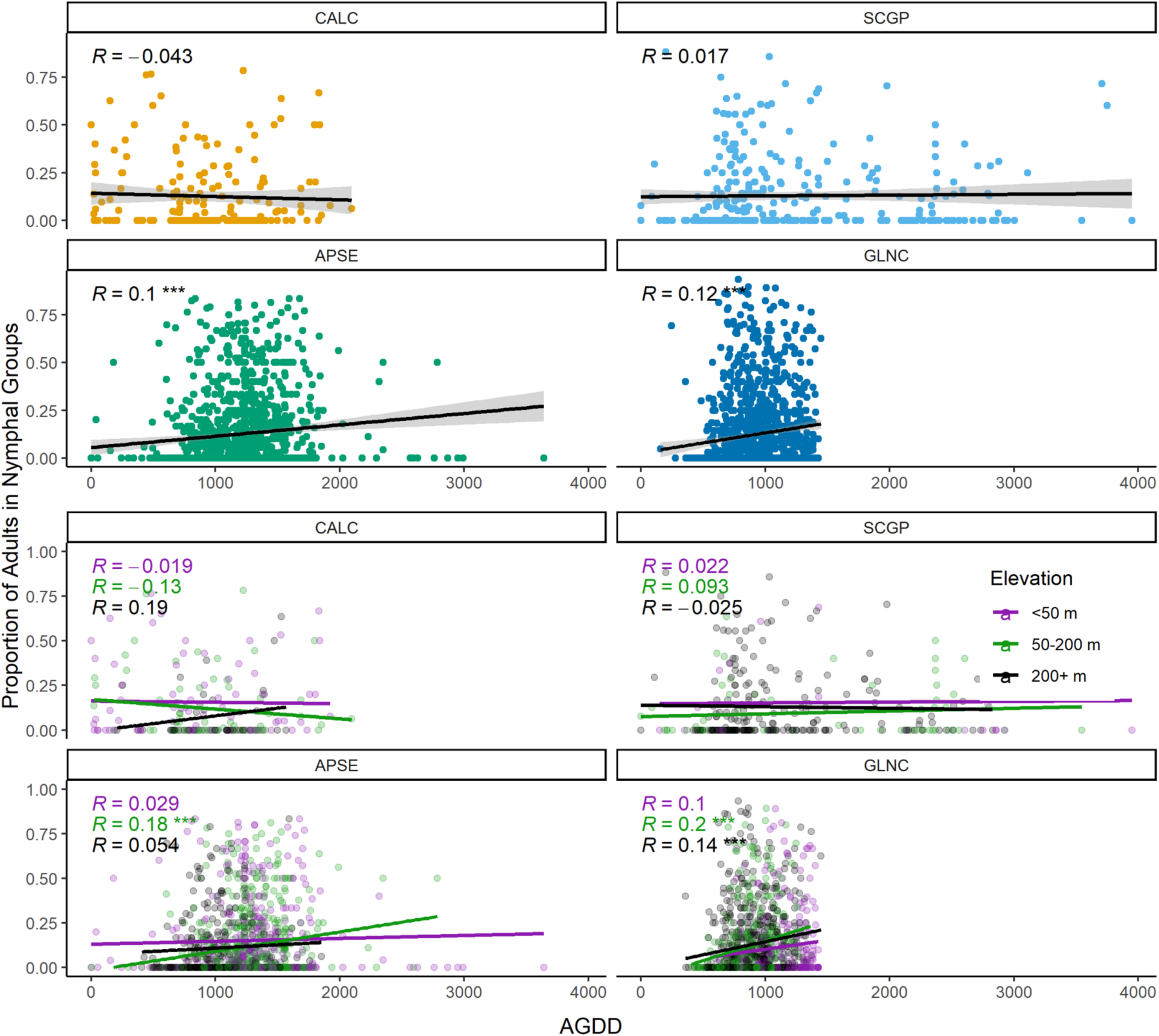
The proportion of *Oncopeltus fasciatus* adults in nymphal groups as a function of the day of the year. Gray area is the standard error. Developmental progressions were further subdivided by elevation (bottom) and Pearson's correlation coefficients (*R*) provided for each elevation category. *** indicated with P < .0001.

Age structure (the proportion of adults within a group) was positively but weakly correlated with AGDD for the APSE (Pearson's correlation coefficient: *R* = .1, *p* = .0002), as well as the GLNC ecoregion (*R* = .12, *p* < .0001) (Figure 6). The proportion of adults in observations from mid (50–200 m) elevations in APSE and GLNC and high (200 + m) elevations in GLNC was positively correlated with AGDD, while observations at lower (<50 m) elevations observations did not show an increase in progression to adults with increasing AGDD (Figure 5). There was no correlation between age structure and AGDD for the other two ecoregions (CALC: *R* = −.04, *p* = .56; SCGP: *R* = .017, *p* = .77; Figure 6). The results for DOY and elevation are presented in Figure S3 and largely followed the results with AGDD. The variability of the developmental progression around this correlation is highly significant, so the explanatory power of these models is quite low, so future work using finer‐grained measures of developmental progression may provide more insight into future studies.

## 4 DISCUSSION

Our study unveiled complexity in the phenological responses of *Oncopeltus fasciatus* life‐history stages to climate variables across the continental range of this insect. Using image‐based citizen science data, we found that the drivers of mating and nymphal phenology differed across ecoregions, with nymphs and adults in eastern and western regions responding to different abiotic variables or in different directions to the same variable. The iNaturalist data were overwhelmingly a reliable source for correctly identified, scientifically beneficial observations for this species. Together, our findings underscore the utility of photodocumented citizen science observations to monitor species interactions, life history, and climate impacts for wide‐ranging species.

### 4.1 Mating

Our assessment across the entire *O. fasciatus* range found that after controlling for the correlation between maximum temperature and daylength, relatively higher temperatures for a given day were associated with later, rather than earlier, mating dates. We interpret this to mean that relatively warmer temperatures expanded the length of the mating season. However, the extent of this seasonal expansion differed by ecoregion. In particular, populations in the CALC ecoregion mated year‐round, but mating occurred earlier when winter precipitation was higher and expanded by 5 days or more for each 1°C relative temperature increase. The importance of winter precipitation to *O. fasciatus* in the CALC region corresponds to recent publications indicating a strong role for precipitation in limiting *Asclepias* distributions in arid regions, though studies of plants point to summer rather than winter precipitation as a driving factor (Lemoine, 2015).

In contrast to phenological responses in CALC, populations in the GLNC ecoregion, where the annual mean temperature was below 27°C, mated 1 to 2 days earlier per 1°C relative temperature increase, suggesting that shorter daylengths prevent a longer expansion of the mating season in northern populations. Our results support calls for investigations of the role of solar seasonality in the physiological responses of plants and insects to climate change (Spence & Tingley, 2020). Climate is often assumed to be the primary factor contributing to phenological processes, but daylength or photoperiod also influences insect physiological processes. *Oncopletus fasciatus* uses photoperiod cues to migrate northward from southern populations in spring and southward from northern populations in fall (Sauer & Feir, 1973). Females enter reproductive diapause in response to shortened daylength prior to migratory flights (Dingle, 1974) and can show phenotypic plasticity related to their ability to reabsorb eggs (Attisano et al., 2013). Early laboratory studies showed that *O. fasciatus* females reared at 27°C did not enter reproductive diapause even under short photoperiods (Dingle, 1974; Dingle et al., 1980). Should temperatures in the GLNC ecoregion increase above 27°C, southward migration may be disrupted in this species.

Previous work (Sauer & Feir, 1973) found similar differences in mating phenology across latitudes, with continuous mating in tropical regions. The mating of *O. fasciatus* in the South Central and Western U.S. in this study extended more than a week later for each 1°C of relative temperature increase. Thus, under future climate warming, the arrival times of migratory individuals from these populations ultimately may dictate mating phenology in northern latitudes. If the delays we observed represent an expansion of the mating season in southern latitudes, then these areas may continue to provide migrants to northern regions at earlier times of the year as well. Should growth and flowering in *Asclepias* advance with climate change (Howard, 2018), northern *O. fasciatus* populations may experience a seasonal expansion in favorable mating conditions. However, if it is a true delay in mating, then northern populations within the GLNC ecoregion may experience a greater mismatch with host plant availability if migrants arrive past peak flowering dates. In the context of potential range restriction, the mating season may remain short, since *O. fasciatus* populations in northern ecoregions seem to be less responsive to temperature, and reports from other studies suggest later flowering times for *A. syriaca* and *A. ovalifolia*, species common in the eastern U.S. and Great Lakes regions, respectively (Dunnell & Travers, 2011).

Our results suggest that species shifting to more northward ranges to adapt to changing climatic conditions may be constrained by photoperiod (Spence & Tingley, 2020). For instance, temperature alone does not facilitate northward expansion for many plant species (Bjorkman et al., 2017), which may threaten the stability of species interactions, particularly for specialist herbivores like *O. fasciatus* (but see Renner & Zohner, 2018). For wide‐ranging compared with more locally distributed herbivorous insects, variation in host plant responses to the same or different climate variables may indirectly mitigate or exacerbate insect responses. Populations in different ecoregions face differences not only in the degree of climate change, degree of local adaptation, and host plant phenology responses but also in host plant losses and in anthropogenic threats to both plants and insects in distinct parts of their full range. In summary, range shifts may allow *Oncopeltus* to maintain thermal tolerance but might alter voltinism, reproductive diapause, and migration behavior for this species.

### 4.2 Nymphs

Responses of nymphs to relative changes in temperature differed substantially over the species' range. Rather than advancing, nymphal presence in California populations was approximately 5 days later for each 1°C of relative temperature increase. As with adults, because nymphs are present all year in the CALC ecoregion, we interpret this to indicate greater numbers of nymphs in later months and an expansion of nymphal feeding season in western regions. These delays were the opposite of nymphal responses in eastern regions, where increases in relative temperature advanced nymphal phenology, highlighting large‐scale and high regional variation in climate sensitivity in this species. Nymphal timing was also delayed by increases in summer precipitation in eastern ecoregions, which may relate to decreased solar insolation during periods of cloud cover.

During development, nymphs rely on seed pod production in *Asclepias* sp. (Miller & Dingle, 1982); milkweed pods may be available continuously in southern climates but are only present seasonally in more northerly regions. Thus, low‐latitude regions sustain year‐round populations, and northern populations fluctuate and are replenished by migrants from the southern regions (Leslie, 1990). Baldwin and Dingle (1986) found that increased temperature decreased maturation time, allowing *O. fasciatus* to mature and mate at an earlier time for populations in the southern latitudes. In our GAMs for nymphal phenology in eastern regions, nymphs were observed approximately 1 day earlier for each 1°C increase in residual temperature. We found only weak positive correlations, however, with increasing AGDDs and rates of nymphal progression, and we found the progressions varied substantially around the correlations (Figure 6 and Figure S3). The use of AGDDs to predict development may not be useful at this larger spatial scale. Further, some of this variability could likely be because of adult migratory behavior. Adults may disperse far from the initial cluster shortly after advancement to adulthood, leading to this metric of progression being quite rudimentary. This approach may, therefore, be better suited to species whether adults and nymphs remain in the same habitat and are not as widely dispersed after maturation. However, despite these limitations, the correlations were significant in both the southeast and northeast ecoregions. Thus, in northern areas where populations are limited by both temperature and daylength, the nymphal development rate is more responsive to climate variables.

In the iNaturalist data, nymphal group sizes were larger in the colder northern latitudes (GLNC ecoregion). Northern populations of *O. fasciatus* have a larger documented nymphal group size, as colder climates compress reproduction into a shorter period synchronous with seed pod availability (Baldwin & Dingle, 1986). Nymphal aggregation can contribute to greater feeding efficiency and increase the rate of juvenile development by assisting juveniles in piercing the pod wall and digesting seeds (Ralph, 1976). We observed nymphal aggregations more often on seed pods and those aggregations on seed pods were larger than those on plant leaves or stems (Figure 2). Nymph clusters disperse when exploring for a new host plant, later re‐aggregating on to feed again in groups (Ralph, 1976). We often observed nymphs on parts of the plant other than seed pods, particularly when leaves and branches were nearby and the cluster was too big to fit on the closed pods available. In fact, our largest nymphal aggregations were those coded as “not on the plant,” suggesting that larger groups may move more often in search of suitable seed pods.

### 4.3 Accuracy of identifications on citizen science observations

Our study demonstrates that iNaturalist data are a reliable source for phenology, species interaction, and life‐history data. This is especially true for organisms similar to *O. fasciatus*, which are commonly photographed, distinctively colored, broadly distributed, and restricted in host use. In our dataset of thousands of observations, the vast majority (98.3%) of research‐grade observations were correctly identified. Even in the much larger set of 22,279 attempts at identifications, *Lygaeus* was consistently the most common taxa mistaken for *O. fasciatus* and about a quarter of incorrect identifications were congeneric species. Misidentifications included Hemiptera with the hallmark red‐on‐black aposematic coloration of the Müllerian mimicry complex that extends across North America (e.g., *Boisea*, *Dysdercus*, *Pyrrhocoris*, *Scantius*, *Sephina*, *Tropidothorax*, *Zelus*) (Berenbaum & Miliczky, 1984; Duffey & Scudder, 1972; Sillén‐Tullberg et al., 1982). The non‐Hemiptera misidentifications were much less common but included *Tetratopes* sp., which also use milkweed (*Asclepias* sp.), or beetles with similar color patterns (*Calopteron*, *Chauliongnathus*, *Liliocerus*). This suggests that citizen scientists, rather than being poor identifiers of insect taxa, can be led astray in predictable patterns or by organisms sharing the same color patterns, habitats, or ecosystems. Patterns in misidentification can guide future uses of iNaturalist data and efforts to improve identifications, as they allow prediction of potentially confounding species, and research users are usually aware of known mimics of target species.

However, our study demonstrates some meaningful limitations in the utility of these citizen science observations. First, the quality and angle of the photos precluded assessment of specific nymphal instar stages. This limitation influenced the degree of specificity our models of nymphal development could attain. Moreover, because photographs capture a single moment in time for these individuals instead of monitoring an individual adult, egg, or nymphal aggregation over time, we do not have specific egg deposition or eclosion dates for nymphal clusters. This prevented us from using more fine‐grained measures of temperature and its impacts on development, which contributed to our weak correlations between growing degree days and development (Figure 6) and to our lower‐than‐anticipated model fits (Table 1). Additionally, we selected *O. fasciatus* because it is a widely photographed, charismatic species on an actively monitored host plant, which increased our sample sizes to be able to assess phenology and life history questions across the range. However, for other less highly photographed species, the limitations in scale and temporal scope may be more substantial and prevent these analyses. Finally, known spatial and temporal biases exist in opportunistic citizen science data (Callaghan et al., 2019; Courter et al., 2013; Tang et al., 2021). Citizen scientists tend to collect data in the warmer months of the year and in more densely populated regions and green spaces near major metropolitan areas. By jointly modeling latitude and longitude to account for spatial structure, we are able to account for some of this limitation, but we recommend cautious interpretation in analyses of this type.

Despite these limitations, citizen science observations documented the location and phenology of *O. fasciatus* and simultaneously documented the mating behavior and the age structure of nymphal groups, as well as interactions with host plants. We have only scratched the surface of opportunities to explore these relationships in citizen science data, even within this species. For example, we did not consider questions related to the sex ratio in observation photos, but many other species may be more readily visually distinguished by sex. Furthermore, we did not differentiate between instar stages in these observations, which would enable finer‐grained investigations of developmental progressions and their relationship to climate variability. Finally, while we included information about the host plant through the nymphal group plant part occupancy, photos were often too close‐up to determine the host plant species identity or fully document the phenology of the host plant. Future work investigating relationships between host plant phenology and insect phenology will be enabled by clear documentation of the identity and phenological status of the host plant as well as the arthropod community. Encouraging documentation of these types of species interaction data in citizen science platforms will further enable future work to capitalize on the wealth of information captured by citizen scientists.

## AUTHOR CONTRIBUTIONS

**Alexis Garretson:** Conceptualization (lead); data curation (lead); formal analysis (lead); investigation (lead); methodology (lead); visualization (lead); writing – original draft (lead); writing – review and editing (lead). **Tedra Cuddy:** Conceptualization (supporting); data curation (supporting); formal analysis (supporting); investigation (supporting); methodology (supporting); writing – original draft (supporting); writing – review and editing (supporting). **Alexandra G. Duffy:** Formal analysis (supporting); validation (supporting); visualization (supporting); writing – original draft (equal); writing – review and editing (equal). **Rebecca E. Forkner:** Conceptualization (supporting); data curation (supporting); formal analysis (supporting); methodology (supporting); project administration (lead); supervision (lead); validation (supporting); visualization (supporting); writing – original draft (equal); writing – review and editing (equal).

## FUNDING INFORMATION

TC gratefully acknowledges the support of the George Mason University Office of Scholarship, Creative Activities, and Research (OSCAR) and the Federal Work‐Study Research Assistant Program. AG was funded by the National Science Foundation Graduate Research Fellowship (DEB No. 1842191).

## CONFLICT OF INTEREST STATEMENT

The authors declare no competing conflicts of interest.

## PERMISSION TO REPRODUCE MATERIALS FROM OTHER SOURCES

None.

## Supporting information


Appendix S1

## Data Availability

The *Oncopeltus fasciatus* occurrence dataset from iNaturalist is available through iNaturalist and as a darwinCore occurrence dataset through GBIF, and the full dataset, including author‐generated annotations and climate data from Daymet, is available with EML metadata in the Environmental Data Initiative Repository under package identifier edi.1326.1 at doi:10.6073/pasta/d710da7ea884535475f1c737b931550f
