# Supplementary material for: Citizen science data reveal regional heterogeneity in phenological response to climate in the large milkweed bug, Oncopeltus fasciatus
Source: Ecol Evol. 2023 Jul 10;13(7):e10213. doi: 10.1002/ece3.10213 (PMC10332934; doi:10.1002/ece3.10213)
Supplement: Supplementary file 1 — Appendix S1 [file ECE3-13-e10213-s001.docx]

**Figure S1.** General additive model of daylength with maximum daily temperature for all 11,224 iNaturalist observations of *Oncopeltus fasciatus*.
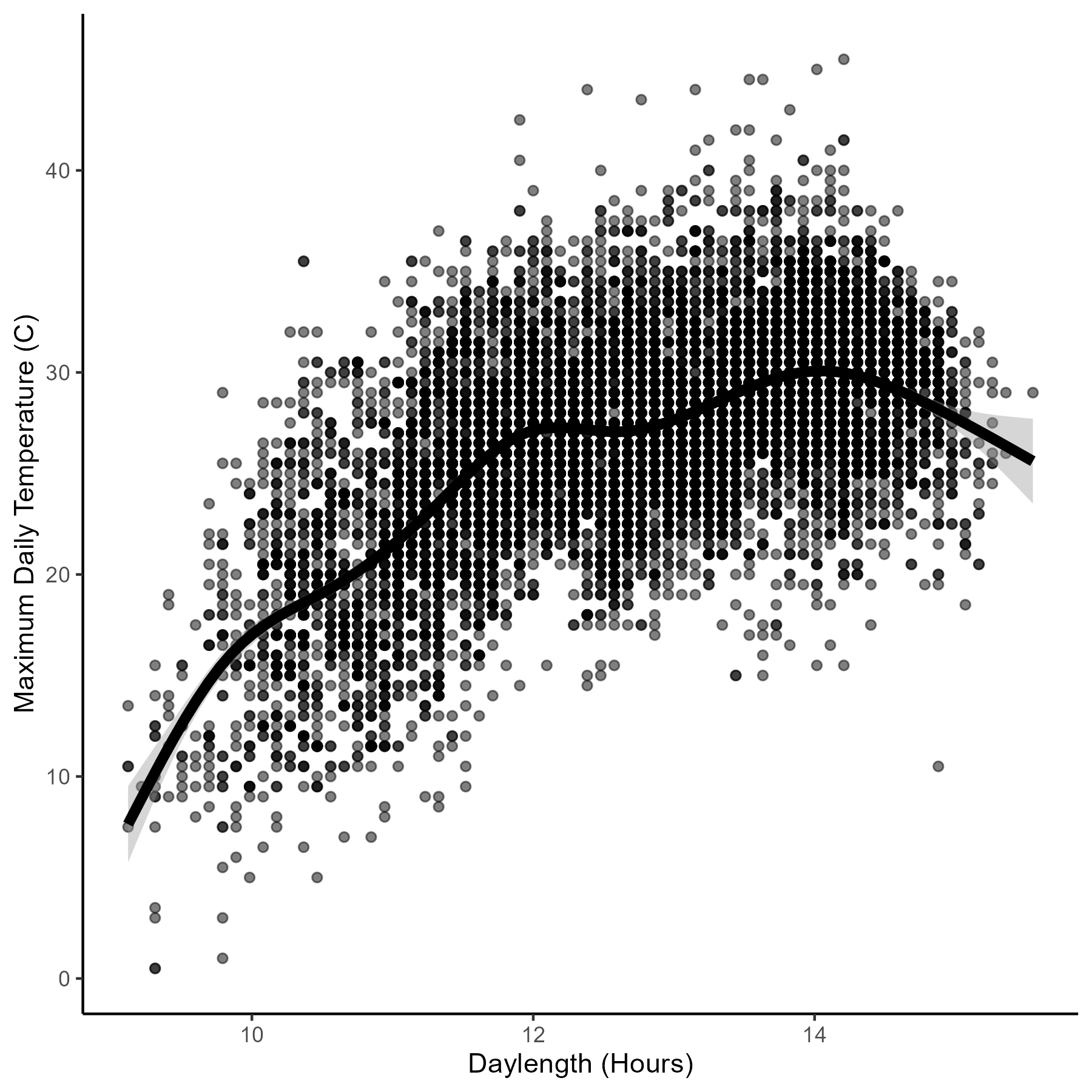


**Figure S2.** Daylength with maximum daily temperature for iNaturalist observations of nymphal groups of *Oncopeltus fasciatus* by ecoregion


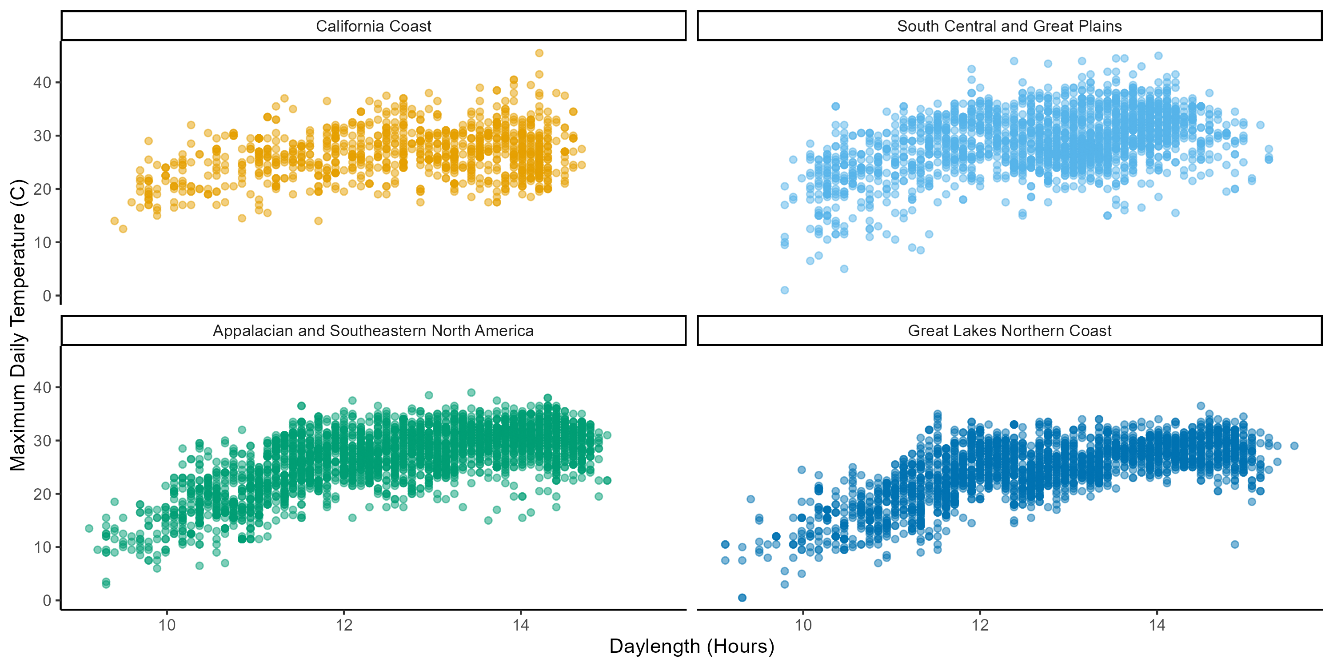


**Figure S3.** The proportion of adult *Oncopeltus fasciatus* insects within nymphal groups. Gray shading represents 95% confidence intervals. The proportion of adult *Oncopeltus fasciatus* insects within nymphal groups for the two ecoregions are also subdivided by elevation (C and D): <50 m (purple), 50 - 200 m (green), and 200+ m (black).

**
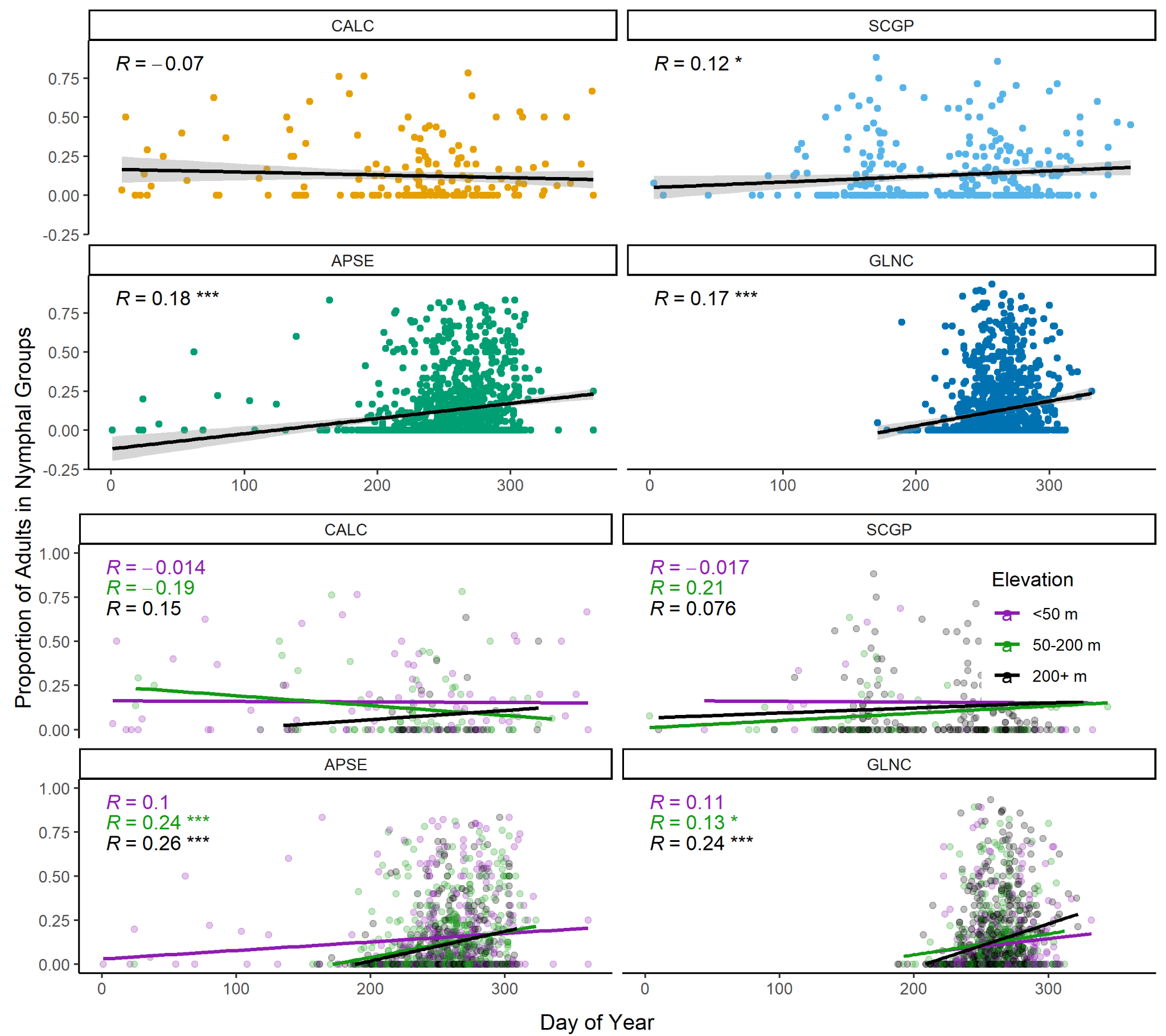
**

**Table S1:**

**Results from a quasibinomial family general linear model with day of year (top) or accumulated growing degree days (AGDD) (bottom), elevation, and their interaction as predictors of the proportion of subadults in nymphal clusters.**

| **DOY** | | | | | | | |
| --- | --- | --- | --- | --- | --- | --- | --- |
| **Model** | **Mean** | **n** | **Model P** | **McFadden's R2** | **Day of Year** | **Elevation** | **Day of Year * Elevation** |
| **CALC** | 0.126±0.186 | 192 | 0.77426 | 2.22% | -0.001 ± 0.002 | -0.002 ± 0.005 | 0.000004 ± 0.000018 |
| **SCGP** | 0.124±0.188 | 302 | 0.65527 | 1.80% | 0.005 ± 0.002 * | 0.002 ± 0.001 | -0.000008 ± 0.000005 |
| **APSE** | 0.127±0.194 | 1212 | 0.00023 | 5.16% | 0.007 ± 0.002 ** | -0.011 ± 0.003 *** | 0.000038 ± 0.000012 ** |
| **GLNC** | 0.124±0.194 | 1220 | 0.0016 | 4.02% | 0.006 ± 0.005 | -0.014 ± 0.006 * | 0.000054 ± 0.000023 * |
| **FULL** | 0.126±0.193 | 2926 | 0.0034 | 1.52% | 0.005 ± 0.001 *** | -0.002 ± 0.002 | 0.000006 ± 0.000006 |
| **AGDD** | | | | | | | |
| **Model** | **Mean** | **n** | **Model P** | **McFadden's R2** | **AGDD** | **Elevation** | **AGDD * Elevation** |
| **CALC** | 0.126±0.186 | 192 | 0.7272 | 2.34% | -0.0003 ± 0.0003 | -0.003 ± 0.002 | 0.000002 ± 0.000002 |
| **SCGP** | 0.124±0.188 | 302 | 0.9518 | 0.38% | 0.0001 ± 0.0002 | 0.0005 ± 0.0006 | -0.0000005 ± 0.0000005 |
| **APSE** | 0.127±0.194 | 1212 | 0.0017 | 1.23% | 0.0004 ± 0.0002 * | -0.001 ± 0.001 | 0.0000009 ± 0.000001 |
| **GLNC** | 0.124±0.194 | 1220 | 0.0204 | 2.59% | 0.0008 ± 0.0005 | -0.0017 ± 0.0023 | 0.000003 ± 0.0000022 |
| **FULL** | 0.126±0.193 | 2926 | 0.2546 | 0.45% | 0.0003 ± 0.0001 ** | 0.000006 ± 0.0004 | -0.0000001 ± 0.0000004 |
